# The effect of the subcritical fluid extraction on the quality of almond oils: Compared to conventional mechanical pressing method

**DOI:** 10.1002/fsn3.1023

**Published:** 2019-06-27

**Authors:** Zhou Qi, Jia Xiao, Liu Ye, Wan Chuyun, Zheng Chang, Li Shugang, Huang Fenghong

**Affiliations:** ^1^ Oil Crops Research Institute, Chinese Academy of Agricultural Sciences of the Ministry of Agriculture, Key Laboratory of Oilseeds Processing, Hubei Key Laboratory of Lipid Chemistry and Nutrition Ministry of Agriculture, Oil crops and Lipids Process Technology National & Local Joint Engineering Laboratory Wuhan China; ^2^ Beijing Advanced Innovation Center for Food Nutrition and Human Health Beijing Technology and Business University Beijing China; ^3^ School of Biological Engineering and Food Hubei University of Technology Wuhan Hubei Province China

**Keywords:** almond oil, bioactive substances, extraction techniques, thermal stability, Xinjiang cultivars

## Abstract

This study investigated the effect of different almond oil extraction techniques, namely, cold‐press extraction (CP), hydraulic press extraction (HP), and subcritical fluid extraction (SFE), on the fatty acid composition, physicochemical properties, bioactive substances, and thermal stability. The results showed that oleic acid and linoleic acid were the main unsaturated fatty acids in almond oil (AO). The overall physicochemical properties of the AO (SFE) had the better oil quality compared to cold‐press extraction and hydraulic press extraction in three kinds of varieties. Almond oil extracted from SFE contained the highest levels of total phenolics (9.58–11.75 mg/100 g), total phytosterols (92.86–244.21 mg/100 g), total tocopherols, and tocotrienols (48.03–55.74 mg/100 g). Meanwhile, the TG/DTG curves showed AO (SFE) were more thermally stable than AO (CP) and AO (HP) consistent with the result of oxidative induction time. Subcritical fluid extraction may be a useful extraction technology to produce high‐quality almond oils in the future.

## 1 INTRODUCTION

Almond seed, is a well‐known nut in the world widely distributed in Mediterranean district, USA and Western China, has been cultivated for thousands of years (Vázquezaraújo, Verdú, Navarro, Martínezsánchez, & Carbonellbarrachina, 2009). As the unique area and climate of almond cultivation in Xinjiang region of western China, it has strong desert sand‐fixing viability. Meanwhile, there is a great potential commercial prospects and economic interest in Xinjiang region.

Almond was consumed as baked goods, original cereals, and confection arise due to their nutritional value, desirable flavor attributes, and high energy density (Xiao et al., 2014). Furthermore, almond is also regards as vegetable oil resource due to its high fat content with high level of unsaturated fatty acid. (Lin et al., 2016). Therefore, another promising approach of almond utilize was a high‐quality oil in edible oil market accept conventional snacks. A plant of studies have focused on the characteristic of almond indicated that the almond oil was benefit for human health, thanks for its special function on reducing the risk of total cholesterol glycemic index, cardiovascular diseases, and so on (Bolling, Dolnikowski, & Blumberg, 2010; Yada, Lapsley, & Huang, 2011). Almond oil was rich in micronutrients, for example tocopherols, sterols, and squalene were 450 μg/g, 2,200 μg/g, and 95 μg/g, respectively (López‐Ortiz et al., 2008; Maguire, O'Sullivan, Galvin, O'Connor, & O'Brien, 2004; Matthäus & Brühl, 2008). These components, seen as conventional antioxidants, contributed to the diversity of physiology, biological, and biochemistry function.

The quality of vegetable oil is mainly determined by the raw materials and the process. The conventional extract methods consist of enzyme‐assisted extraction (Winkler, Foidl, Gübitz, Staubmann, & Steiner, 1997), mechanical pressing, solvent extraction (Chen, Cheng, Ching, Hsiang, & Chang, 2012; Sayyar, Abidin, Yunus, & Muhammad, 2009). Generally, solvent extraction can affect the degradation of active‐oxidative compounds and the products safety (Chan & Ismail, 2009). However, residual solvent is hard to remove absolutely. Hence, mechanical pressing is considered as the preferred method to extract oil from seed (Achten et al., 2010). For instance, cold‐pressed technology has been promoted for a long time because its advantages such as without solvent, low press temperature compared with the traditional process. Until now, a new extraction technology, subcritical fluid extraction (SFE), was based on the low polarity fluid extraction. It also can overcome the defects of the conventional organic solvent extraction and expeller pressing methods (Liu et al., 2008). In applied subcritical fluids exaction solvents, n‐butane is regarded as the most practical fluid thanks for its lower critical pressures and temperature, meanwhile its extraction ability for lipophilic substances. SFE is also attracting increasing attention because of the shorter extraction time, high yield, and environmentally responsibility (Jimenez, Masson, Barriga, Chavez, & Robert, 2011; Wang, Liu, Chen, & Wang, 2011; Xia, You, Li, Sun, & Suo, 2011; Xu et al., 2011). SFE has been widely reported for plant seed extractions such as canola oil and sunflower oil (Jimenez et al., 2011), flaxseed oil (Pradhan, Meda, Rout, Naik, & Dalai, 2010; Zanqui et al., 2015), camellia seed oil (Miao et al., 2013), and wheat germ oil (Shao, Sun, & Ying, 2008). Nevertheless, the comparison of above three techniques ( SFE, CP, and HP) was limited in application of almond oil.

Except for the extraction method, genotypes were also a main source for chemical traits in plant oil (Maestri et al., 2015). Macronutrients and micronutrients in several genetic almond kernel and almond oil were reported in California, USA, Egypt, Greece, Turkey, and so on (Askin, Balta, Tekintas, Kazankaya, & Balta, 2007; Bolling et al., 2010; Nanos, Kazantzis, Kefalas, Petrakis, & Stavroulakis, 2002; Yada et al., 2011). However, a little study was focus on the Xinjiang region. During our preliminary experiment, three kinds of typical varieties from 9 genotype varieties in Xinjiang region has been selected derived from excellent quality and a certain scale of planting area. A investigation has been carried out for cold‐pressed oil extracted from 9 types of almond cultivars, indicated that SC‐9, SC‐ZP, and SC‐TX cultivars have high quality in terms of nutrition and oxidation stability (Zhou, et al, 2017).

A complete investigation needs to conduct based on three extract methods (cold‐press extraction, hydraulic press, and subcritical fluid extraction) with respect to chemical composition, physicochemical characteristics, and thermal stability. The results reported here may serve as a guide to produce high‐quality almond oil to improve the inherent chemical components and conservation ability in process of almond.

## 2 MATERIAL AND METHODS

### 2.1 Materials

Three types of local major almond cultivars (SC‐9, SC‐ZP, and SC‐TX) in Xinjiang region was collected in 2015. Those samples were chosen for the experiment because Xinjiang region was the biggest production region, and the total amount of SC‐9 SC‐ZP and SC‐TX occupied one third of Xinjiang region. Moisture in almond kernel was ranged from 4% to 5%.

### 2.2 Chemicals

Analytical grade hexane, ethanol, chloroform, and sodium hydroxide were supplied by Shanghai Guo Yao Medicine Chemical Reagent Co., Ltd. (Shanghai, China). Methanol and acetonitrile (chromatographic grade) were purchased from Merck Chemical Co., Ltd (Darmstadt, Germany). High purity helium was supplied by Ming Hui Gas Co., Ltd. (Wuhan, China). All the standards (α, β, γ, δ‐tocopherols and α, β, γ, δ‐tocotrienols standards, β‐sitosterol, stigmasterol, campesterol and brassicasterol, sinapic acid) were purchased from Chromadex Co., Ltd. (Irvine, CA, USA).Fatty acid methyl ester (C_8_‐C_30_) was derived from Sigma‐Aldrich Co. (Shanghai, China). N‐Butane (food grade, purity 99.99%) was purchased from Henan subcritical Biotechnology Manufacturing Co., Ltd (Hunan, China).

### 2.3 Extraction procedures

#### 2.3.1 Cold‐press extraction (CP)

Almond kernel was extracted by cold‐press with a small proportion of hull in order to increase the squeezing pressure. Oil was obtained using a spiral screw press (CA59G, Komet Co. Stuttgart, Baden‐Wuerttemberg, Germany) with a 5‐mm restriction die. A screw speed of 20 rpm was kept in the whole pressing process. The screw press was first run empty for 5 min without any material to raise the screw press barrel temperature. In order to maintain the screw press barrel temperature to the indoor temperature, the press barrel was heated via an electrical resistance‐heating ring (Martínez, Penci, Marin, Ribotta, & Maestri, 2013). The cold‐pressed oils were centrifuged. The oils were protected and stored under N_2_ gas at −4°C prior to analysis.

#### 2.3.2 Hydraulic press extraction (HP)

Approximately 1,000 g of sample was placed in the pressing chamber. Almond oil was obtained by hydraulic pressure extraction (YD32‐30, Changzhou, China) and pressures up to 25 MPa as Subrotoel method (Subroto, Manurung, Heeres, & Broekhuis, 2015). Operation temperature of hydraulic press machine was range from 30°C to 105°C. Total pressing time was 10 min. Hydraulic pressed oils were centrifuged at 8,000 rpm for 15 min. The extracted oils were protected and stored under N_2_ gas at −4°C prior to analysis.

#### 2.3.3 Subcritical fluid extraction (SFE)

Almond kernel was smashed, through a 60 mesh screen. Subcritical fluid extraction unit was customized by Henan of subcritical Biotechnology Manufacturing Co., Ltd. About 250 g samples were placed by subcritical liquid extraction with butane as a solvent. The pressure in the extraction tank was reduced to −0.09 MPa, and a certain volume of butane was added to the extraction. After the extraction, the extraction liquid is introduced into the separation tank to open the compressor for solvent recovery and dissolve. When the pressure of the separation tank and the extraction tank falls below 0.3 MPa, the vacuum pump is opened until the pressure of the two tanks was reduced to −0.09 MPa. Almond kernel was extracted by two times, and final collected oil was centrifuged and was protected and stored under N_2_ gas at −4°C prior to analysis.

### 2.4 Analytical methods

#### 2.4.1 Total fat, protein, and moisture content of almond seeds

Almond oils were investigated in terms of crude fat (method GB/T 5512–2008), crude protein (method GB/T 14489.2–2008), and moisture (method GB/T 14489.1–2008).

#### 2.4.2 Amino acids composition of almond seeds

Amino acid analysis of almond seed was made according to method GB/T 5009.124–2003 using a L‐8900 amino acid analyzer (Hitachi, Japan). Almond samples were first hydrolyzed to convert proteins into their constituent amino acids. Almond sample was put into hydrolysis tubes, 6M HCl was added, evacuated, and filled with nitrogen thrice, and then hydrolyzed at 110°C for 22 hr. The amino acids were analyzed by monitoring the absorption at 570 nm and 440 nm.

#### 2.4.3 Physicochemical properties of almond oils

Physicochemical properties of AO were analyzed including oil yield (extract oil weigh/ almond weigh, %), oil moisture (method GB/T 5528–2008), acid value (AV, method GB/T 5530–2008), peroxide value (PV, method GB/T 5538–2008), iodine value (IV, method GB/T 5532–2008), and saponification value (SN, method GB/T 5534–2008).

#### 2.4.4 Fatty acid composition of almond oils

Fatty acid composition was determined by GC‐FID after transmethylation with methanolic potassium hydroxide. 0.05 g oil sample was weighed and saponified in 0.5 M sodium methoxide. The fatty acids methyl esters were analyzed by 7890A (Agilent, Santa Clara, CA, USA) provide with a polar capillary column HP‐INNOWX (30 m × 0.25 mm × 0.25 mm film thickness, Supelco, Bellefonte, PA, USA). Temperature programming was from 210℃ and increased to 260°C at the rate of 20°C/min, and ultimately hold for 5.0 min. The injector volume was 1 ml. Split ratio was 80:1. A reference fatty acid standards (C_8_‐C_30_) was synchronously separated to qualitative analysis by comparing their retention times. The final result was expressed as percentage of total peak areas.

#### 2.4.5 Total phenol content

Total phenolic content was detected according to Yang Mei et al. method (Yang et al., 2013).

#### 2.4.6 Phytosterols content

0.03 g oil sample was mixed with 3 ml of 2 M KOH in 95% ethanol. Then it was shaken in a water bath at 90°C for 15 min, adding 2 ml of water and 1.5 ml of hexane. The mixture was centrifuged at 5,000 rmp for 3 min, and the organic phase was separated for further analysis. A nonpolar DB‐5 column (15 m × 0.32 mm id, 0.1 μm film thickness) was performed. The flow rate was set at 1.5 ml/min with nitrogen as the carrier gas. The injection volume was 1 ml, and the split ratio was 10:1. The oven temperature was programmed as follows: 180°C to 243°C at 3°C/min and hold for 0.5 min. Then, the temperature was increased at a rate of 50°C/min to a final temperature of 340°C, and keep for 0.5 min. Identification of the individual phytosterol was accomplished by peak times and standard compounds.

#### 2.4.7 Tocopherol and tocotrienols content

0.05 g oil sample was weighed into a glass tube, added into 10 ml mixed solution (ethyl acetate: cyclohexane was 1:1). Take 3 ml solution and put into GPC, then collect objectives and volume to 2 ml with methanol. High‐performance liquid chromatography with C18 column conditions as follows: moving phase: The ratio of methanol and water was 98:2, column temperature was 30°C, UV detection wavelength was 296 nm, and the injection volume was 10 μL. Qualitative use retention time and quantification use external standard.

#### 2.4.8 Rancimat test and DSC analysis

Rancimat test in almond was according to Matthäus method (Matthäus & Brühl, 2008) with a slight modification It was determined at 110°C, under a constant air flow (20 L/h). Thermogravimetric (TG) technique on a thermogravimetric analyzer (Q2000, TA Instruments Inc., USA) was used to evaluate the thermal stability under normal air atmosphere (100 ml/min) conditions. The weighed samples (5 mg) weighed in open solid fat index (SFI) aluminum pans (T70529, TA Instruments) and were put into open alumina crucibles, then cooling to 10°C from room temperature. Heating program: The temperature was ranged from 10°C to 350°C at a heating rate of 10°C/min. Thermal oxidation temperature was determined in three times.

### 2.5 Statistical analysis

Analysis of variance (ANOVA) and Duncan's multiple range tests were carried out by using SPSS (Statistical Product and Service Solutions) 18.0 software version. All experiments were performed in quintuplicate. All the figures with means and standard deviations for all values were computed in origin 8.0.

## 3 RESULTS AND DISCUSSION

### 3.1 Characterization of fat, protein, and moisture in almond seeds

**Figure 1:**
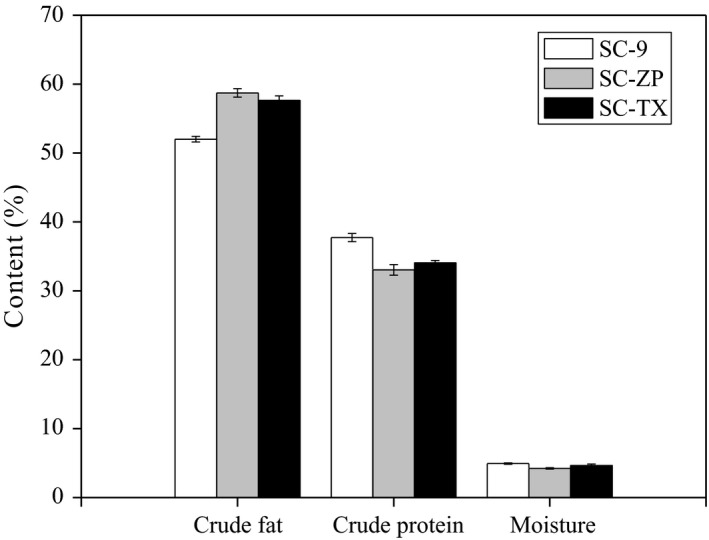
Composition (% dry matter) of three almond seeds

The main ingredient in almond seed was fat, protein, and carbohydrates. The composition of almond may be influenced due to cultivar and season (Vázquezaraújo et al., 2009). From Figure 1, the fat content in almond seeds was 52.00%, 58.72%, and 57.62% in SC‐9, SC‐ZP, and SC‐TX, respectively. Maestri et al.(2015) have reported that total fat contents in almond kernels were ranged from 48.0% to 57.5%, and the value for oil content at the average of 50%. SC‐ZP has the highest fat content indicating high quality of Xinjiang region. Meanwhile, protein content was 33.04%, 37.73%, and 34.04% in SC‐9, SC‐ZP, and SC‐TX. The moisture content was 4.93%, 4.24%, and 4.65% in almond seeds at SC‐9, SC‐ZP, and SC‐TX, respectively. Martínez et al.(2013) indicated that the mean value for protein and moisture could be around 25.56% and 4.50% in almond kernels.

### 3.2 Amino acids in almond seeds

The amino acid compositions in almond were shown in Table 1. 16 amino acids were detected, and the most abundant one was Glu, followed by Asp then Arg. The total amino acids were accounted for 20.51, 21.80, and 20.42 g/100 g protein in SC‐9, SC‐ZP, and SC‐TX, respectively. There was no significant difference between SC‐9 and SC‐TX. The exceptional thing is the contents of individual amino acids were all higher than the other ones. These results agree to the Zhou et al. studies (Zhou et al., 2017), where found no significant difference of total amino acid content among varieties of hempseed oils.

**Table 1:** Amino acids composition (g/100 g protein) in almond seed

Protein	SC−9	SC‐ZP	SC‐TX
Asp	2.46 ± 0.12	2.70 ± 0.01	2.35 ± 0.04
Thr	0.59 ± 0.02	0.67 ± 0.02	0.62 ± 0.01
Ser	0.89 ± 0.03	0.97 ± 0.03	0.90 ± 0.03
Glu	5.48 ± 0.21	5.65 ± 0.07	5.46 ± 0.08
Gly	1.14 ± 0.05	1.24 ± 0.02	1.14 ± 0.05
Ala	0.94 ± 0.03	1.04 ± 0.01	0.95 ± 0.03
Cys	0.42 ± 0.01	0.43 ± 0.05	0.46 ± 0.04
Val	0.99 ± 0.02	1.08 ± 0.07	1.00 ± 0.02
Met	0.21 ± 0.01	0.21 ± 0.01	0.23 ± 0.01
Iso	0.81 ± 0.08	0.86 ± 0.04	0.79 ± 0.05
Leu	1.37 ± 0.06	1.48 ± 0.04	1.36 ± 0.04
Tyr	0.65 ± 0.03	0.69 ± 0.01	0.65 ± 0.01
Phe	0.96 ± 0.04	1.01 ± 0.03	0.93 ± 0.02
Lys	0.50 ± 0.01	0.57 ± 0.02	0.56 ± 0.02
His	0.44 ± 0.03	0.45 ± 0.01	0.43 ± 0.03
Arg	2.00 ± 0.04	2.07 ± 0.02	1.93 ± 0.01
Pro	0.66 ± 0.05	0.68 ± 0.05	0.66 ± 0.05
Total AA	20.51 ± 0.66	21.80 ± 0.77	20.42 ± 0.37

### 3.3 Physicochemical properties of almond oils from different extraction methods

**Figure 2:**
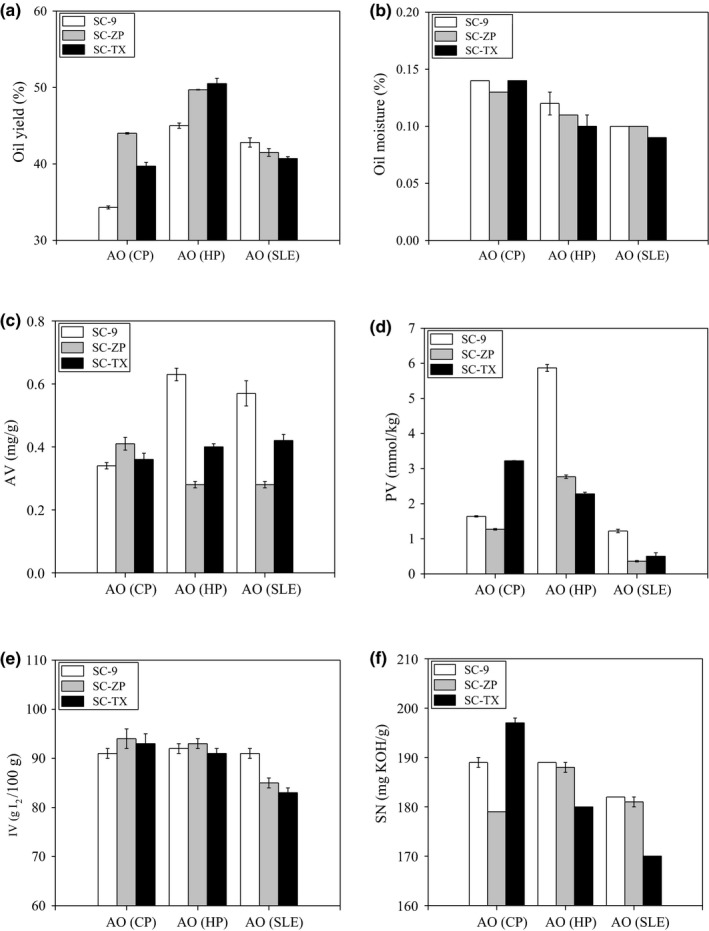
Chemical properties of almond oils: Oil yield (a), Oil moisture (b), AV (c), PV (d), IV (e), SN (f)

Fat content in seed and extraction methods play roles one extraction properties (Aladić et al., 2014). Fat content of 58.72% in SC‐ZP was highest in all samples. Its corresponding oil yield 44.05% for CP, 49.71% for HP, and 41.52% for SFE in SC‐ZP, respectively. The best extraction method for oil yield was HP, followed by SFE then CP in all samples. Pradhan et al. (Pradhan et al., 2010) found oil yield of supercritical CO_2 _extract oil (35.31%) was higher in comparison with the screw press process (25.55%). Liu et al. (Liu et al., 2008) showed that the high oil yield was attained based on n‐butane solvent subcritical fluid extraction. Part of oil moisture was exceed the limit in oil, for instance 0.14% for CP, 0.12% for HP in SC‐9, 0.13% for CP, 0.11% for HP in SC‐ZP, 0.14% for CP in SC‐TX. However, the almond oil ranged from SFE opposed a moisture under safe storage condition. Oils from cold‐pressed method may need to dehydrate free moisture. Oil moisture content in almond oil from SFE was lowest, suggesting the AO from SFE may more stable during storage due to low moisture content (Onyeike et al., 1995). AV, PV, IV, and SN of the extracted almond oils from this study are also shown in Figure 2. AV indicates the level of free FA as a result of lipase activity in oil. AV were ranged from 0.28 mg/g to 0.57 mg/g. PV, another parameter for evaluating oil quality, in the samples studied here PV were 1.22 mmol/kg for SFE in SC‐9, 0.36 g/100 g in SC‐ZP, 0.50 mmol/kg in SC‐TX. PV value from SFE was lower than that in CP and HP, which confirmed good oxidative stability of AO from SFE. Meanwhile, IV (g of I_2_ per 100 g of oil) showed the levels of unsaturation and potential oxidative sensitivities of the oils (Moodley, Kindness, & Jonnalagadda, 2007). IV was ranged from 83 g/100 g to 94 g/100 g. IV value in AO from SFE was lower than that in CP and HP. These values were lower than in Zizyphi spinosi semen obtained by supercritical fluid extraction (IV = 109.7) (Wang et al., 2011). IV of Camellia seed oil was 83.20 according to Miao report (Miao et al., 2013), also extracted from SFE. SN value reflects the average molecular weight in oil (Wang et al., 2011). SN was ranged from 179 to 197 mg/g, for CP, from 180 to 189 mg/g for HP, and from 170 to 182 mg/g for SFE. SN value in AO from SFE was lower than that in CP and HP. Those results of SN obtained from the almond oils were in good agreement with Moodley et al. (2007), who reported the SN value of regular almond was 182.5 mg/g. However, these values are higher than kernel oils (SN = 108.19 mg/g) reported by Farhoosh and Tavakoli (2008).

### 3.4 Fatty acid composition in almond oils from different extraction methods

The FA composition of AO has been extensively researched for different species (Yada et al., 2011). FA compositions of almond oils are shown in Table 2. The oils contained palmitic acid, palmitoleic acid, stearic acid, oleic acid, linoleic acid, and linolenic acid. The most abundant FA in AO is oleic acid, followed by linoleic acids, representing 72%–76% and 15%–20% of the total FA content, respectively. The levels of total UFA in all almond oil were similar. These results agree with the previous studies, where fatty acid content in flaxseed oil from solvent extraction and SFE extract had no significant difference (Zanqui et al., 2015). However, there were slight variations between the PUFA and MUFA contents (Table 2). Main major monounsaturated fatty acid (MUFA) among all studied species was oleic acid (C18:1) ranging from 72.92% to 75.92%. Main polyunsaturated fatty acids (PUFA) present were linoleic acid (C18:2) ranging from 15.64% to 19.23%. The content of MUFA was found to be 76.54%, 73.58%, and 76.55% in the AO of SC‐9 (CP), SC‐9 (HP), and SC‐9 (SFE), and the MUFA in AO (SFE) was similar to that in AO (CP) but was higher than that in AO (HP). The amount of PUFA in AO by SFE was similar to CP, but was lower than HP, suggesting the HP method was more efficient in PUFA. Han et al. (Han, Cheng, Zhang, & Bi, 2009) found content of UFA in safflower seed oil obtained by SC‐CO_2_ extraction (91.57%) was higher than those in oils obtained expeller pressing (89.72%) and solvent extraction (88.33%). Pradhan et al. (2010) found the percentage of UFA in supercritical CO_2_ extract flaxseed oil (88.7%) was higher in comparison with the screw press (84.3%).The ratio of MUFA to PUFA is an important reference indicator for oil stability in unsaturated oils (Kodad & Socias, 2008; Sathe, Seeram, Kshirsagar, Heber, & Lapsley, 2008). In the current study, SFE and CP had the highest ratio of MUFA to PUFA (4.86), corresponding HP had the lowest MUFA to PUFA ratio (3.96), suggesting AO by SFE have high stability. Kodad and Socias (2008) have found high stability of highest ratio of MUFA to PUFA in almond kernels.

**Table 2:** Fatty acid composition in almond oils (relative content, %)

Methods	Fatty acid	C16:0	C16:1	C18:0	C18:1	C18:2	C18:3	∑PUFA	∑MUFA	∑UFA	∑MUFA/∑PUFA
AO (CP)	SC−9	6.54 ± 0.02	0.62 ± 0.01	1.17 ± 0.01	75.92 ± 0.06	15.64 ± 0.06	0.1 ± 0.00	15.74 ± 0.12	76.54 ± 0.07	92.28 ± 0.19	4.86
	SC‐ZP	6.45 ± 0.02	0.46 ± 0.01	1.40 ± 0.01	72.57 ± 0.07	19.00 ± 0.04	0.1 ± 0.00	19.10 ± 0.04	73.03 ± 0.08	92.13 ± 0.12	3.82
	SC‐TX	6.49 ± 0.01	0.54 ± 0.01	1.40 ± 0.02	73.43 ± 0.06	18.09 ± 0.03	0.1 ± 0.00	18.19 ± 0.03	73.97 ± 0.0.07	92.16 ± 0.10	4.07
AO (HP)	SC−9	6.74 ± 0.01	0.66 ± 0.02	1.08 ± 0.01	72.92 ± 0.06	18.38 ± 0.03	0.2 ± 0.00	18.58 ± 0.03	73.58 ± 0.08	92.16 ± 0.11	3.96
	SC‐ZP	6.36 ± 0.01	0.52 ± 0.01	1.31 ± 0.02	72.36 ± 0.05	19.23 ± 0.01	0.2 ± 0.00	19.43 ± 0.01	72.88 ± 0.06	92.31 ± 0.07	3.75
	SC‐TX	6.38 ± 0.01	0.57 ± 0.01	1.17 ± 0.01	73.74 ± 0.09	18.01 ± 0.03	0.1 ± 0.00	18.11 ± 0.03	74.31 ± 0.10	92.42 ± 0.14	4.10
AO (SFE)	SC−9	6.58 ± 0.01	0.66 ± 0.02	1.11 ± 0.03	75.89 ± 0.09	15.64 ± 0.04	0.1 ± 0.00	15.74 ± 0.04	76.55 ± 0.11	92.29 ± 0.15	4.86
	SC‐ZP	6.85 ± 0.02	0.48 ± 0.02	1.21 ± 0.03	72.61 ± 0.07	18.74 ± 0.05	0.1 ± 0.00	18.84 ± 0.05	73.09 ± 0.09	91.93 ± 0.14	3.88
	SC‐TX	6.55 ± 0.02	0.55 ± 0.01	1.20 ± 0.06	73.61 ± 0.07	17.88 ± 0.05	0.2 ± 0.00	18.08 ± 0.05	74.16 ± 0.08	92.24 ± 0.13	4.10

### 3.5 Bioactive substances in almond oils from different extraction methods

#### 3.5.1 Total phenols

Total phenols content and range in the AO was investigated shown in Table 3. Total phenols amount ranged from a minimum of 4.71 mg/100 g to maximum of 11.75 mg/100 g. The total phenols in AO extracted by SFE derived from SC‐9 (11.75 mg/100 g), SC‐ZP (10.59 mg/100 g), and SC‐TX (9.58 mg/100 g) were higher than that by CP derived from SC‐9 (5.51 mg/100 g), SC‐ZP (4.71 mg/100 g), and SC‐TX (8.40 mg/100 g) and by HP derived from SC‐9 (7.62 mg/100 g), SC‐ZP (8.92 mg/100 g), and SC‐TX (7.45 mg/100 g), respectively. The results suggested that the SFE was more effective in extracting of total phenol from almond seed. These results agreed the results of Mezzomo, Mileo, Friedrich, Martinez, and Ferreira (2010), who found the AO may get a better retention of phenolic compounds by SFE method compared with conventional techniques. Similar phenomenon also was observed by Shao, Liu, Fang, and Sun (2015), who found the content of total phenols in tea seed oils extracted by supercritical fluid extraction (78.7 mg/kg) was higher than that by cold‐press extraction (65.9 mg/kg).

**Table 3:** Bioactive substances in almond oils

	AO (CP)			AO (HP)			AO (SFE)		
	SC−9	SC‐ZP	SC‐TX	SC−9	SC‐ZP	SC‐TX	SC−9	SC‐ZP	SC‐TX
Total phenol (mg/100 g)	5.51 ± 0.21	4.71 ± 0.31	8.40 ± 0.54	7.62 ± 0.1	8.92 ± 0.23	7.45 ± 0.42	11.75 ± 0.37	10.59 ± 0.69	9.58 ± 0.71
Phytosterols (mg/100 g)									
Brassicasterol	9.91 ± 0.52	n.d.	n.d.	n.d.	n.d.	n.d.	5.66 ± 0.24	n.d.	2.40 ± 0.31
Campesterol	5.12 ± 0.30	n.d.	n.d.	n.d.	n.d.	n.d.	22.2 ± 0.90	3.36 ± 0.51	8.20 ± 0.62
Stigmasterol	31.0 ± 1.61	n.d.	n.d.	n.d.	n.d.	n.d.	60.1 ± 3.57	6.50 ± 0.82	23.74 ± 0.83
β‐sitosterol	82.07 ± 4.54	58.05 ± 2.54	56.80 ± 3.11	60.91 ± 3.05	19.37 ± 1.54	23.57 ± 1.44	156.10 ± 4.70	83.00 ± 3.71	74.51 ± 3.70
Total Phytosterols	128.13 ± 6.97	58.05 ± 2.54	56.80 ± 3.11	60.91 ± 3.05	19.37 ± 1.54	23.57 ± 1.44	244.21 ± 9.14	92.86 ± 5.04	108.85 ± 5.46
Tocopherol and tocotrienols (mg/kg)									
α‐tocopherol	33.29 ± 2.70	30.31 ± 2.42	33.46 ± 1.85	17.64 ± 0.13	25.89 ± 0.17	21.93 ± 0.31	30.28 ± 0.52	30.7 ± 0.61	36.49 ± 0.82
β + γ‐tocopherol	5.86 ± 0.23	6.48 ± 0.45	4.48 ± 0.18	3.24 ± 0.14	7.70 ± 0.15	6.50 ± 0.24	5.48 ± 0.31	8.03 ± 0.42	8.89 ± 0.51
δ‐tocopherol	n.d.	n.d.	n.d.	n.d.	0.31 ± 0.04	0.38 ± 0.02	0.25 ± 0.02	0.45 ± 0.05	0.54 ± 0.05
α‐tocotrienols	0.41 ± 0.01	0.39 ± 0.03	0.29 ± 0.01	0.31 ± 0.02	0.25 ± 0.01	0.18 ± 0.01	0.27 ± 0.03	0.27 ± 0.02	0.24 ± 0.01
β + γ‐ tocotrienols	0.21 ± 0.01	0.21 ± 0.01	0.23 ± 0.02	n.d.	n.d.	n.d.	n.d.	n.d.	n.d.
δ‐tocotrienols	n.d.	n.d.	0.78 ± 0.01	n.d.	n.d.	n.d.	n.d.	n.d.	n.d.
Total vitamin E	45.28 ± 2.95	42.1 ± 2.91	47.64 ± 2.07	28.81 ± 3.39	43.07 ± 0.60	36.44 ± 0.58	48.03 ± 0.88	50.04 ± 1.00	55.74 ± 1.39

#### 3.5.2 Phytosterols

Phytosterols were found in plant and marine materials and known for their decrease the low‐density lipoprotein (LDL) cholesterols (Racette et al., 2010). Phytosterols were detected and the most abundant one was β‐sitosterol in both AO samples and ranged from 60.91 mg/100 g (HP) to 156.10 mg/100 g (SFE) (Table 3). The β‐sitosterol in AO extracted by SFE derived from SC‐9 (156.10 mg/100 g), SC‐ZP (83.00 mg/100 g), and SC‐TX (74.51 mg/100 g) were higher than that by CP derived from SC‐9 (82.07 mg/100 g), SC‐ZP (58.05 mg/100 g), and SC‐TX (56.86 mg/100 g),which derived from HP. In addition, the total phytosterols in AO extracted by SFE derived from SC‐9 (244.21 mg/100 g), SC‐ZP (92.86 mg/100 g), and SC‐TX (108.85 mg/100 g) were higher than that cold‐pressing technology derived from SC‐9 (122.9 mg/100 g), SC‐ZP (58.0 mg/100 g), and SC‐TX (56.8 mg/100 g), significantly higher than SC‐9 (60.9 mg/100 g), SC‐ZP (19.3 mg/100 g), and SC‐TX (23.5 mg/100 g) in HP, which suggested that the SFE method was more effective in extracting of β‐sitosterol and total phytosterols from almond seed. The extracted total content was nearly twofold compared to cold‐pressed ones. This phenomenon is consistent with the results reported by Shao et al.(2015), who showed the total phytosterol contents in tea seed oils extracted by supercritical fluid extraction (3,820 mg/kg) was higher than that in by cold‐press extraction (3,388 mg/kg).

#### 3.5.3 Tocopherol and tocotrienols

In collected almond, eight vitamin E compounds were detected. The four individual tocopherol compounds(α, β, γ, and δ) have a saturated 16‐carbon phytol on the side chain; however, the corresponding tocotrienols (α, β, γ, and δ) have three double bonds (Kornsteiner, Wagner, & Elmadfa, 2006). The α‐tocopherol seems to be the most important contributor to both the radical scavenging capacity and the oxidative stability of almond kernel (Kornsteiner et al., 2006). AO studied in the present work showed α‐tocopherol was the most prevalent tocopherol in AO and it ranged from 17.64 to 36.49 mg/kg (Table 3). Interestingly, the content of a‐tocopherol from SFE in SC‐TX and SC‐ZP was higher than that in CP and HP, which suggested that the SFE method was more effective in extracting of a‐tocopherol from almond seed. This phenomenon is consistent with the results of the Mariod et al. reports (Mariod, Matthäus, & Ismail, 2011), who showed that a‐tocopherol content (40.07 mg/100 g) of kenaf seed oil extracted by SFE was higher than Soxhlet (19.76 mg/100 g), and antioxidant capacity of flaxseed hull oils extracted from SFE (1.18) was higher than cold‐press (0.61) (Oomah & Sitter, 2009). β‐ and γ‐tocopherol are minor vitamin E components in SC‐ZP and SC‐TX and the mean amounts in descending order were SFE > HP>CP. Accordingly, Bozan and Temelli (Bozan & Temelli, 2002) have also reported the content of β‐ and γ‐tocopherol in flaxseed oil obtained by SFE (73.9 mg/kg) was higher than that obtained by HP (53.7 mg/kg).

Sight traces of δ‐tocopherol (<5 mg/kg AO) were found in SC‐9, SC‐ZP, and SC‐TX. The total vitamin E in almond oil extracted by SFE derived from SC‐9 (48.03 mg/kg), SC‐ZP (50.04 mg/kg), and SC‐TX (55.74 mg/kg) were higher than that by CP derived from SC‐9 (45.28 mg/kg), SC‐ZP (42.1 mg/kg), and SC‐TX (47.64 mg/kg) and by HP derived from SC‐9 (28.81 mg/100 g), SC‐ZP (43.07 mg/100 g), and SC‐TX (36.44 mg/100 g), respectively. Almond oil extracted by SFE exerted better oxidative stability. Previous studies have demonstrated almonds rich in tocopherols and polyphenolic compounds, and these bioactive compounds can protective against lipid oxidation (Bolling et al., 2010).

### 3.6 Thermal stability of almond oil from different extraction methods

From the comparison of micronutrients, total phenolic, and phytosterol contents in almond oil from SFE were significantly higher than those in CP and HP and may contribute to the different process technology. Consequently, the oxidative stability of almond oil was measured using the Rancimat method. This is a commonly used method for comparing the oxidative stabilities of fats and oils. Temperature (110°C) and a constant air flow (20 L/h) were usually chosen as suitable parameters for determining IP (induction period) value (García‐Lomillo, González‐SanJosé, Pino‐García, Rivero‐Perez, & Muñiz, 2014; Parras, Martínez‐Tomé, Jiménez, & Murcia, 2007; Tańska, Mikołajczak, & Konopka, 2018). The IP values at 110°C of AO were found to be 6.50 hr, 1.45 hr, and 7.09 hr in SC‐9 (CP), SC‐9 (HP), and SC‐9 (SFE), respectively, 6.47, 4.28, and 6.77 in SC‐ZP (CP), SC‐ZP (HP), and SC‐ZP (SFE), respectively, 6.26 hr, 3.00 hr, and 6.41 hr in SC‐TX (CP), SC‐TX (HP), and SC‐TX (SFE), respectively (Table 5). Interestingly, the IP values of almond oil in descending order all were SFE > CP>HP, suggesting that the almond oil from SFE has better oxidation stability compared to that from CP and HP. By analyzing the correlation between different active substance and oxidation induction time, Table 4 showed total phytosterols, tocopherol, and tocotrienols have significant correlation with oxidation induction time (*p* < 0.01). However, there is no significant correlation with total phenol and induction time.

**Table 4:** Correlation analysis between bioactive compounds and induction time

	Induction time (IP)
Tocopherol and tocotrienols	*r* = 0.864** *p* < 0.01
Total phytosterols	*r* = 0.560** *p* < 0.01
Total phenol	*r* = 0.220 *p* > 0.05

** Correlation is significant at 0.01 level.

**Figure 3:**
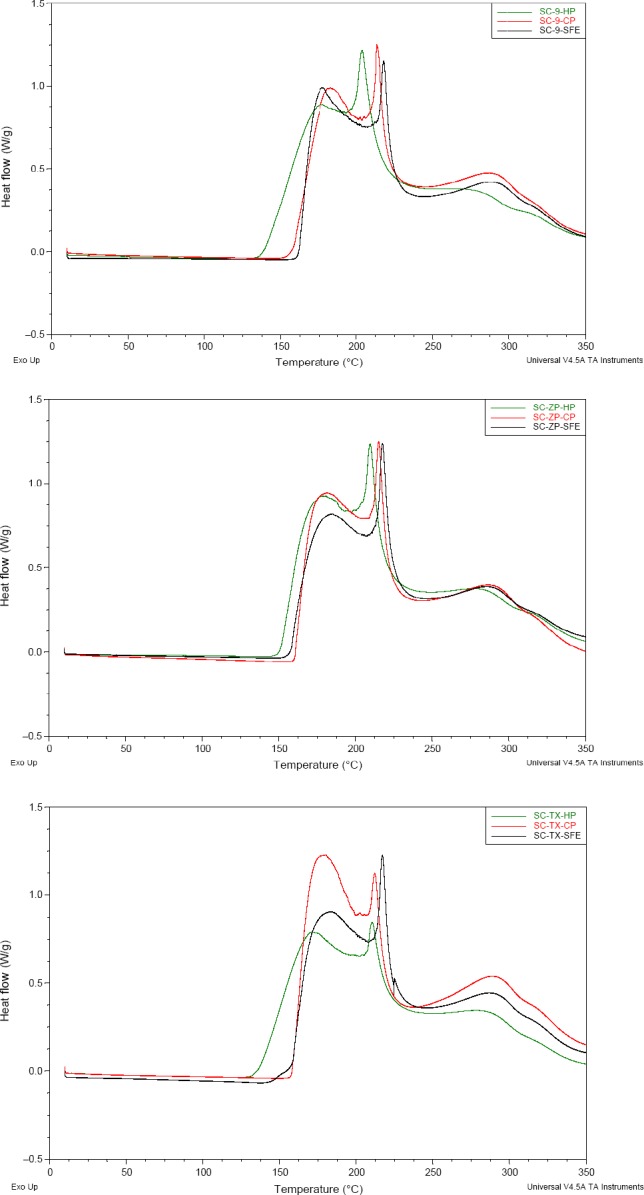
DSC of almond oil in different varieties and technology

In addition, oxidation characteristic of almond oil was assessed by differential scanning calorimetry (DSC). DSC testing revealed three step exothermic effects in almond (Figure 3). These peaks could be considered as cross‐linking level. Oxidation temperature of almond oil started from 150.43 to 160.11°C, within temperatures reported for edible oils from 130 to 180°C (Litwinienko, Daniluk, & Kasprzycka‐Guttman, 1999). Mean onset and oxidation temperatures were 122.35–123.01°C and 157.65–160.11°C, respectively, higher than that in CP and HP (Table 5). Oxidation temperature of first peak was similar to those of flash and smoke points. High kinetic stability in almond oil from SFE characterized by DSC revealed excellent thermal stability, contributed to the high content bioactive compounds of almond oil from subcritical fluid extraction.

**Table 5:** Thermal stability of almond oil by DSC

	AO (CP)			AO (HP)			AO (SFE)		
	SC−9	SC‐ZP	SC‐TX	SC−9	SC‐ZP	SC‐TX	SC−9	SC‐ZP	SC‐TX
IP (h)	6.50 ± 0.12	6.47 ± 0.30	6.26 ± 0.31	1.45 ± 0.25	4.28 ± 0.45	3.00 ± 0.10	7.09 ± 0.12	6.77 ± 0.43	6.41 ± 0.45
Onset	120.31	121.36	120.96	118.62	119.63	119.00	122.35	123.47	123.01
Oxidative temperature (^o^C)	154.59	157.49	158.52	150.43	151.25	155.94	157.88	160.11	157.65
Peak1 (^o^C)	182.26	184.85	179.70	177.82	178.87	171.32	183.28	180.79	183.67
Peak2 (^o^C)	205.06	217.64	212.10	203.82	209.56	210.38	213.47	215.16	217.14
Peak3 (^o^C)	276.21	286.07	288.73	273.03	277.18	278.37	287.24	286.61	286.25
Peak 4 (^o^C)	320.03	319.56	318.95	315.69	318.14	318.99	317.43	319.67	319.46

## 4 CONCLUSIONS

Oleic acid and linoleic acid were the dominant unsaturated fatty acids in almond oil. From conventional index, the overall physicochemical properties of the AO (SFE) had the best oil quality due to PV, IV, and SN in three extraction methods. Almond oil extracted from SFE contained the highest levels of total phenolics (9.58–11.75 mg/100 g), total phytosterols (92.86–244.21 mg/100 g), total tocopherol and tocotrienols (48.03–55.74 mg/100 g) compared to those from CP and HP. The total phytosterols were highest in SC‐9, simultaneously. SC‐TX has abundant total tocopherol and tocotrienols. Meanwhile, the TG/DTG curves showed AO (SFE) were more thermally stable than AO (CP) and AO (HP) consistent with the result of oxidative induction time. By analyzing the correlation between different active components and oxidation induction time, total phytosterols, tocopherol, and tocotrienols showed significant correlation with oxidation induction time (*p* < 0.01). However, there is no significant correlation with total phenol and induction time. The oil from subcritical fluid extraction no need for refining. In all, the subcritical fluid extraction may be a useful extraction technology to produce high‐quality almond oils in the future.

## CONFLICT OF INTEREST

There is no conflict of interest in this paper.

## ETHICAL APPROVAL

All authors were actively involved in the work leading to the manuscript and will hold themselves jointly and individually responsible for its content. There is no conflict of interest in this paper. Human or animal testing is unnecessary in our study.
